# Determination of LMF Binding Site on a HSA-PPIX Complex in the Presence of Human Holo Transferrin from the Viewpoint of Drug Loading on Proteins

**DOI:** 10.1371/journal.pone.0084045

**Published:** 2014-01-02

**Authors:** Zohreh Sattar, Mohammad Reza Saberi, Jamshidkhan Chamani

**Affiliations:** 1 Department of Biochemistry and Biophysics, Faculty of Sciences, Mashhad Branch, Islamic Azad University, Mashhad, Iran; 2 Medical Chemistry Department, School of Pharmacy, Mashhad University of Medical Sciences, Mashhad, Iran; University of Quebect at Trois-Rivieres, Canada

## Abstract

Holo transferrin (TF) and the natural complex of human serum albumin and protoporphyrin IX (HSA-PPIX) are two serum carrier proteins that can interact with each other. Such an interaction may alter their binding sites. In this study, fluorescence spectroscopy, as well as zeta potential and molecular modeling techniques, have been used to compare the complexes (HSA-PPIX)-LMF and [(HSA-PPIX)-TF]-LMF. The K_a1_, K_a2_, values of (HSA-PPIX)-LMF and [(HSA-PPIX)-TF]-LMF were 1.1×10^5^ M^−1^, 9.7×10^6^ M^−1^, and 2.0×10^4^ M^−1^, 1.8×10^5^ M^−1^, respectively, and the n_1_, n_2_ values were respectively 1.19, 1.53 and 1.17, 1.65. The second derivative of the Trp emission scan of (HSA-PPIX)-LMF exhibited one negative band at 310 nm, whereas for the [(HSA-PPIX)-TF]-LMF system, we observed one negative band at 316 nm indicating an increase in polarity around Trp. The effect of TF on the conformation of (HSA-PPIX)-TF was analyzed using three-dimensional fluorescence spectroscopy. The phase diagram indicated that the presence of a second binding site on HSA and TF was due to the existence of intermediate structures. Zeta potential analysis showed that the presence of TF increased the positive charges of the HSA-PPIX system. Site marker experiments revealed that the binding site of LMF to HSA-PPIX changed from Sudlow's site IIA to Sudlow's site IIIB in the presence of TF. Moreover, molecular modeling studies suggested the sub-domain IIIB in HSA as the candidate place for the formation of the binding site of LMF on the (HSA-PPIX)-TF complex.

## Introduction

Human serum albumin (HSA) is a principal extracellular protein with a high concentration in blood plasma (40 mg/ml or 0.6 mM) [1]. It is a globular protein composed of three structurally similar domains (I, II, and III), each containing two subdomains (A and B) and stabilized by 17 disulfide bridges [2], [3]. Aromatic and heterocyclic ligands have been found to bind with two hydrophobic pockets in subdomains IIA and IIIA, namely site I and site II [4]. Seven binding sites are localized for fatty acids in subdomains IB, IIIA, IIIB and at the subdomain interfaces. The multiple binding sites underlie the exceptional ability of HSA to interact with many organic and inorganic molecules and make this protein an important regulator of intercellular fluxes as well as of the pharmacokinetic behavior of many drugs [5]–[8].

Another most plentiful protein in blood plasma is TF. This substance consists of two homologous lobes, each of which is composed of two domains, responsible for transport of metal ions and minerals. TF has 8 Trp residues and is stabilized by 19 intra-chain disulfide bonds. The direct interaction of drugs with TF has been widely studied [9]–[12].

Lomefloxacin (LMF) is a third-generation member of quinolone antibiotics fluorinated in position 6 and bearing a piperazinyl moiety in position 7, capable of penetrating well into cells [13]. LMF is very active against both Gram (+) and Gram (−) bacteria through inhibition of their DNA gyrase and is widely used for the clinical treatment of severe systematic infections, such as soft tissue infection, typhoid fever, bone and joint infections, prostatitis blood poisoning and sinusitis [13].

Porphyrins, as prosthetic groups of enzymes and carrier proteins, are important for several functions. For example, porphyrins participate as electron transporters in oxidation reduction processes of cells, in oxygen transport and storage [14]–[16]. One of the most common natural sources of porphyrin is the iron-free form of heme called protoporphyrin IX [17]. Concentrations of PPIX in normal cells have been found to be low whereas they are high in tumor cells. This is due to the fact that the enzyme ferrochelatase that converts protoporphyrin IX to heme has been found to be reduced in cancerous cells [18].

Based on these characteristics, with increasing amounts of PPIX in the blood, the probability of HSA-PPIX formation increases accordingly. Previous studies [19] have shown that blood proteins interact with each other, and that in the case of cancer amounts of the (HSA-PPIX)-TF complex are increased. This is the main reason to study the interaction of LMF with the (HSA-PPIX)-TF system and investigate the effect of TF in complex. In our previous work, [19] it was demonstrated that LMF bound to site IIA of HSA in the presence of PPIX.

In the present work, the binding parameters of LMF to the (HSA-PPIX)-TF complex have been characterized by fluorescence quenching, zeta potential and molecular modeling techniques. Several studies have been performed to investigate the binding properties related to the secondary structural changes of (HSA-PPIX)-TF, the quenching mechanism, the specific binding site and binding patches. This work should provide a deeper understanding of the transport of LMF. Furthermore, the LMF binding site of HSA-PPIX was investigated in the absence and presence of TF to determine the structural contents of the (HSA-PPIX)-TF complex as well as the binding affinity of LMF to both proteins in separate and complex states.

## Materials and Methods

### Chemicals

HSA, TF, protoporphyrin IX (PPIX), lomefloxacin (LMF) and potassium phosphate were purchased from Sigma Chemical Co. and used without further purification. Dimethyl formamide (DMF), sodium carbonate, ethylene diamine tetra-acetic acid (EDTA), ethanol and sodium hydroxide were obtained from Merck Chemical Co. (Germany). Visking dialysis tubing was procured from Scientific Instrument Center Limited (SIC, Eastleigh, UK). Double-distilled water was used throughout the experiments.

### Sample preparation

HSA-PPIX (4.5×10^−3^ mM) and TF (4.5×10^−3^ mM) were dissolved in 50-mM phosphate buffer solution at pH 7.4. LMF at 0.05 mM was obtained by diluting a stock solution of LMF (0.5 mM) in 50-mM phosphate buffer. A 2.0-ml protein solution (the concentrations of proteins were kept fixed at 4.5 ×10^−3^ mM) was titrated by addition of 10-µl volumes of 0.05 mM LMF. Samples were measured at room temperature. HSA was labeled with PPIX according to Ref. [20]: 3.5 mg of PPIX was dissolved in 100 µl DMF and injected as five aliquots of 20 µl to 2 ml of phosphate buffer containing 200 µM HSA at an interval of 15 min under vigorous stirring. The mixture was stirred vigorously for 1 h after which a mild dialysis was carried out against phosphate buffer for 4.5 h to remove the unreacted PPIX. [21]–[23]

### Fluorescence spectroscopy

Fluorescence measurements were performed on an F-2500 spectrofluorimeter (Hitachi, Japan) with a 150-W Xenon lamp, a 1.0-cm quartz cell and a thermostat bath. The width of both the excitation slit and emission slit was set at 5.0 nm. The operation software automatically corrected the spectral scan for the photomultiplier characteristics. Furthermore, fluorescence intensities were corrected for inner filter and dilution effects before any data analysis was carried out. The excitation was set at 280 and 295 nm, and the emission was collected in the range of 300–600 nm (excitation of Trp and Tyr).

### Three-dimensional fluorescence spectroscopy

Three-dimensional fluorescence spectroscopy were performed with an FP-6200 spectrofluorimeter (Jasco, Japan) equipped with a 150-W Xenon lamp at room temperature. A 1-cm quartz cuvette with four optical windows was used for the analyses. Emission scans were performed from 220 to 500 nm, with steps of 5 nm, and excitation wavelengths from 220 to 500 nm at intervals. The detector was set to high sensitivity. The slit width for excitation and emission was 5 nm, and the experiments were performed under the same conditions as for the fluorescence measurements.

### ‘Phase diagram’ method of fluorescence

The ‘phase diagram’ method of fluorescence is an extremely sensitive technique for the accurate detection of unfolding/refolding intermediates of proteins [24], [25]. The essence of this method is to create a graph of I(λ_1_) versus I(λ_2_), where I(λ_1_) and I(λ_2_) are the fluorescence intensity values measured at wavelengths λ_1_ and λ_2_, respectively, under different experimental conditions for a protein undergoing structural transformations. Since the fluorescence intensity is the extensive parameter, it can describe any two-component system by a simple relationship [25]. 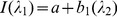
(1)


Where 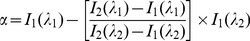
(2) and 
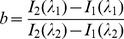
(3)


Here, I_1_ (λ_1_) and I_2_ (λ_1_) are the fluorescence intensities of the first and second components, respectively, measured at wavelength λ_1_, whereas I_1_ (λ_2_) and I_2_ (λ_2_) are those of respectively the first and second components measured at wavelength λ_2_. When applied to protein unfolding, Eq. 1 predicts that the dependence I_1_(λ_1_) = f(I(λ_2_)) will be linear if changes in the protein environment lead to the all-or-none transition between two different conformations. Alternatively, if the transition from the initial to the final state follows a “three-state” or “multi-state” model with the formation of one or several intermediate states, the dependence I_1_(λ_1_) = f(I(λ_2_)) must be nonlinear and will contain two or more linear portions. Each linear portion describes an individual all-or-none transition [21], [22].

### Time-resolved fluorescence

Time-resolved fluorescence decays were recorded on a time-correlated single photon counting ARCUS Fluorometer (LKB, Turku, Finland), with the excitation wavelength at 295 nm and the emission wavelength at 345 nm. The data was fitted to bi-exponential functions after deconvolution of the instrumental response function by an iterative reconvolution approach with the DAS6 decay analysis software utilizing reduced χ and weighted residuals as parameters for goodness of fit. The average fluorescence lifetime (τ) for the bi-exponential iterative fittings was calculated from the decay times and the relative amplitudes (α) using the following equation: 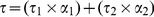
(4)


### Zeta potential measurements

Particle size, polydispersity and zeta potential measurements were conducted on a Malvern Zetasizer. The measurements were performed at ambient temperature. The Henry equation was used to calculate zeta-potentials from measurements of electrophoretic mobility [26]. In this calculation, we assumed the viscosity of the solution to be the same as water.

### Molecular modeling

The docking calculations of LMF's association with HSA-PPIX and TF were undertaken using the Autodock4 program. The crystal structures of HSA-PPIX and TF were retrieved from the RCSB Protein Data Bank (PDB entry: 1n5u and 1suv, respectively). The protein-protein docking program HEX v.5.1, was subsequently used to examine probable modes of interaction between HSA-PPIX and TF. To model the effect of LMF on the interaction between (HSA-PPIX)-TF, the complexes of (HSA-PPIX)-TF were applied and the best docking results were further investigated with WebLab-ViewerLite, Molegro Molecular Viewer and Swiss pdb-Viewer 4.

## Results and Discussion

### Fluorescence quenching

**Figure 1:**
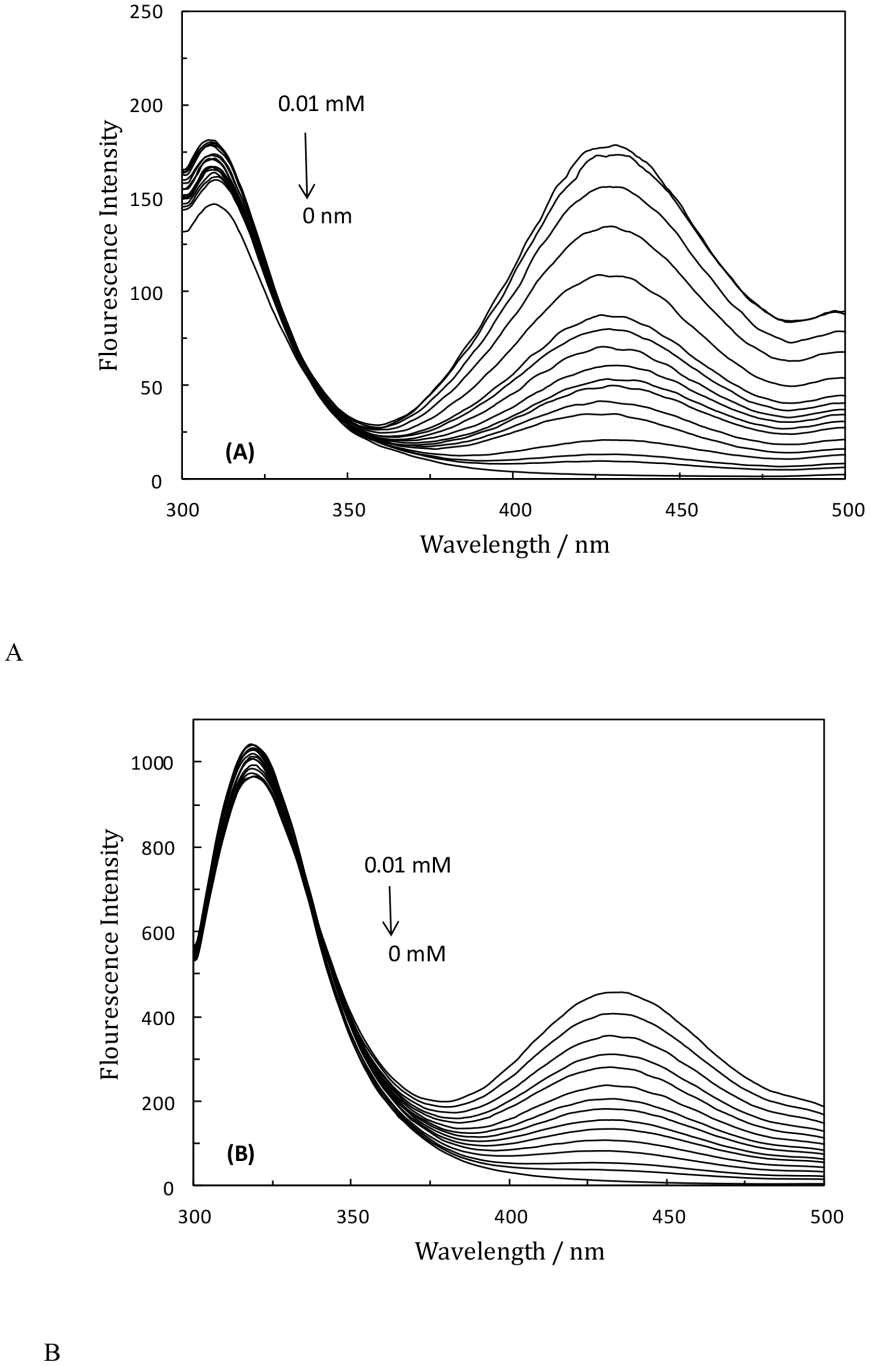
Fluorescence emission spectra of the protein–drug systems in the presence of various concentrations of LMF for the (HSA–PPIX) system (A) and (HSA–PPIX)-TF system (B), λ_ex_ = 280 nm.

When HSA is excited at 285 nm, it radiates strong intrinsic fluorescence at 334 nm. This phenomenon can be explained by the sole Trp residue located at the 214th position of the chain and the Try residues in HSA [27]. The intrinsic fluorescence of HSA is very sensitive and when local surroundings of HSA are altered slightly, the intrinsic fluorescence becomes obviously weakened. Factors such as protein conformational transition, bio-molecule binding, denaturation, etc. are responsible for this weakening [27]. To investigate the effect of TF on the binding of LMF to the (HSA-PPIX)-TF complex, steady-state fluorescence spectra of (HSA-PPIX), shown in Fig. 1A, and of (HSA-PPIX)-TF, displayed in Fig. 1B, were run and showed that the fluorescence intensity of the two systems became consistently weakened when increasing the LMF concentration.

**Figure 2:**
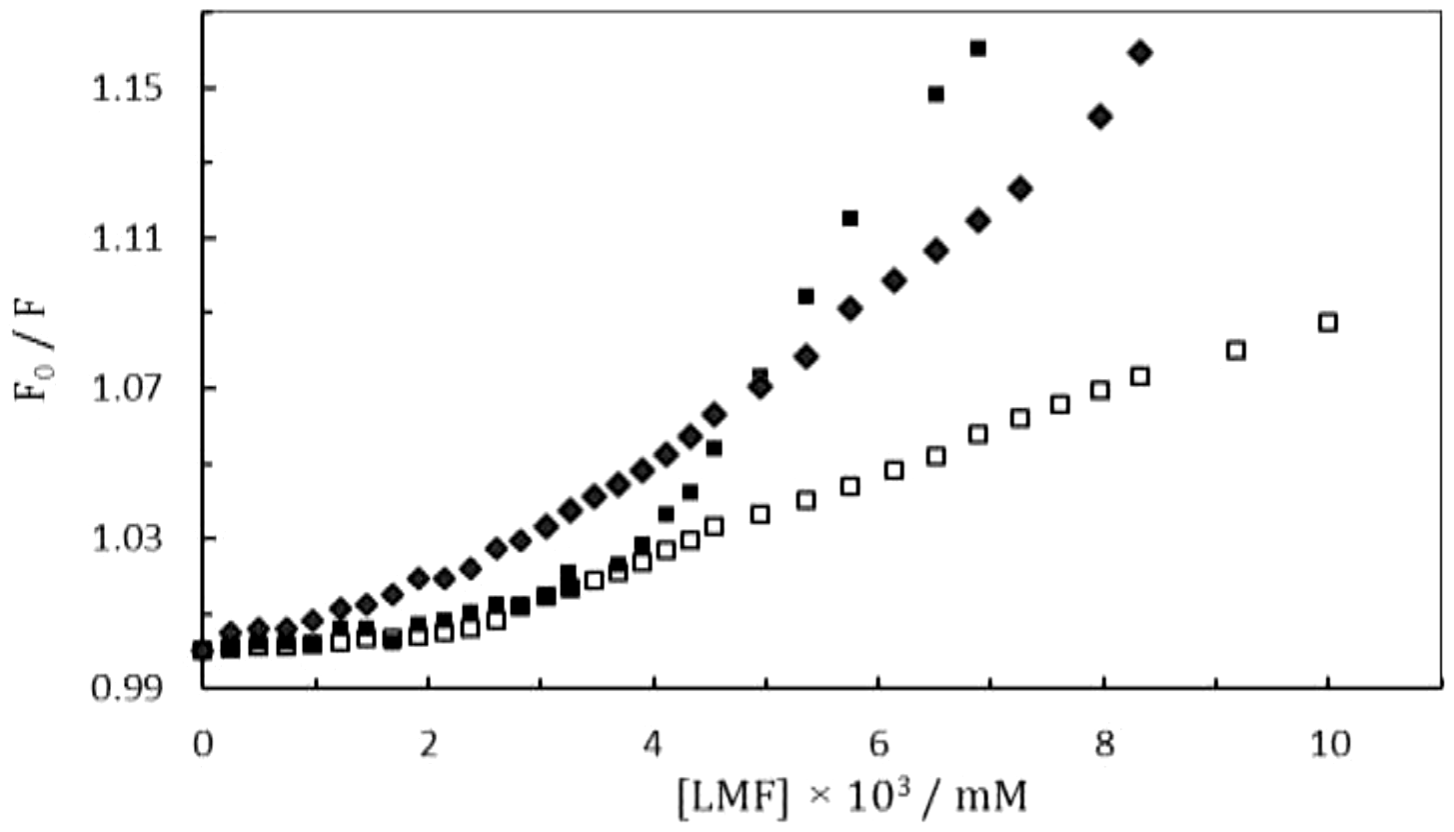
Stern-Volmer quenching plot of TF (filled square), HSA-PPIX (filled lozenge), and complex of TF-(HSA-PPIX) open square, with various concentrations of LMF, following excitation at 280 nm, [LMF] = 0.05 mM, [(HSA-PPIX)-TF] = [HSA-PPIX] = [TF] = 4.5×10^−3^ mM.

The obtained data was subjected to Stern-Volmer analysis, as shown in Fig. 2. In certain situations, the Stern-Volmer plot presented negative divergences from linearity, following a hyperbolic-like function. In general, these situations result from the existence of more than one class of fluorophores, with varying K_sv_ values [28]. The K_sv_ value showed that the rate constant of protein quenching procedure initiated by LMF was greater than the k_q_ of the scatter procedure with the biopolymer (2×10^10^ L mol^−1^ s^−1^) [29]. This suggests that the main cause of quenching was the formation of a complex through a static mechanism as opposed to the dynamic one. Based on free spreading of LMF molecules, we can assume that the dynamic mechanism had a minor effect on the fluorescence quenching.

According to Table 1, there were two different K_sv_ values demonstrating two types of binding affinities. The affinity of the first set of binding sites was low while it increased in the second set, and the affinity of LMF to the protein increased for larger values of K_sv_. Moreover, LMF displayed less binding to the (HSA-PPIX)-TF complex than to HSA-PPIX. In fact, in the presence of TF, the binding affinity of LMF to HSA-PPIX decreased leading to local perturbations of the LMF binding site on the (HSA-PPIX)-TF complex.

**Table 1:** Stern-Volmer quenching constants of the various complexes with LMF at λ_ex_ = 280 nm.

Sample	λ_ex_ = 280 nm				
	K_SV_/M^−1^	k_q_/M^−1^s^−1^	n	K_a_	_R_
TF-LMF	4.61×10^4^	4.61×10^12^	2.54	3.4×10^5^ M^−1^	0.99
	4.56×10^3^	4.56×10^11^	0.29	2.33×10^5^ M^−1^	0.99
(HSA-PPIX)-LMF	9.65×10^3^	9.65×10^11^	1.19	1.1×10^5^ M^−1^	0.99
	2.29×10^4^	2.29×10^12^	1.53	9.7×10^6^ M^−1^	0.99
[(HSA-PPIX)-TF]-LMF	2.36×10^3^	2.36×10^11^	1.17	2.0×10^4^ M^−1^	0.99
	1.08×10^4^	1.08×10^12^	1.65	1.8×10^5^ M^−1^	0.99

### Time-resolved fluorescence

Fluorescence lifetime measurements serve as a sensitive parameter for exploring the quenching mechanism between drugs and proteins; they provide one of the best parameters to help us differentiate between static and dynamic processes [30]. In order to further demonstrate the quenching mechanism, in the absence and presence of LMF, the fluorescence lifetime of (HSA-PPIX)-TF in phosphate buffer (pH 7.4) was determined. The results are tabulated in Table 2. The average fluorescence lifetime was 0.9673 ns with LMF and 0.9576 ns without it, indicating that the fluorescence quenching was essentially a static mechanism. Therefore, both steady-state and time-resolved measurements hinted to the occurrence of static fluorescence quenching caused by specific interactions, mainly ground-state complex formations and a significant energy transfer between the drug and (HSA-PPIX)-TF.

**Table 2:** Time-resolved fluorescence data of the (HSA-PPIX) TF and (HSA-PPIX) TF/drug complexes (λ_ex_ = 295 nm, λ_em_ = 345 nm, pH 7.4, T = 298 K).

System	τ_1_/(ns)	α_1_	τ_2_/(ns)	α_2_	τ (ns)	χ^2^
(HSA-PPIX) TF	1.972	0.7569	5.783	0.3673	3.642	0.9673
[(HSA-PPIX) TF] LMF	1.942	0.7640	6.027	0.3731	3.729	0.9576

### Second derivative fluorescence

**Figure 3:**
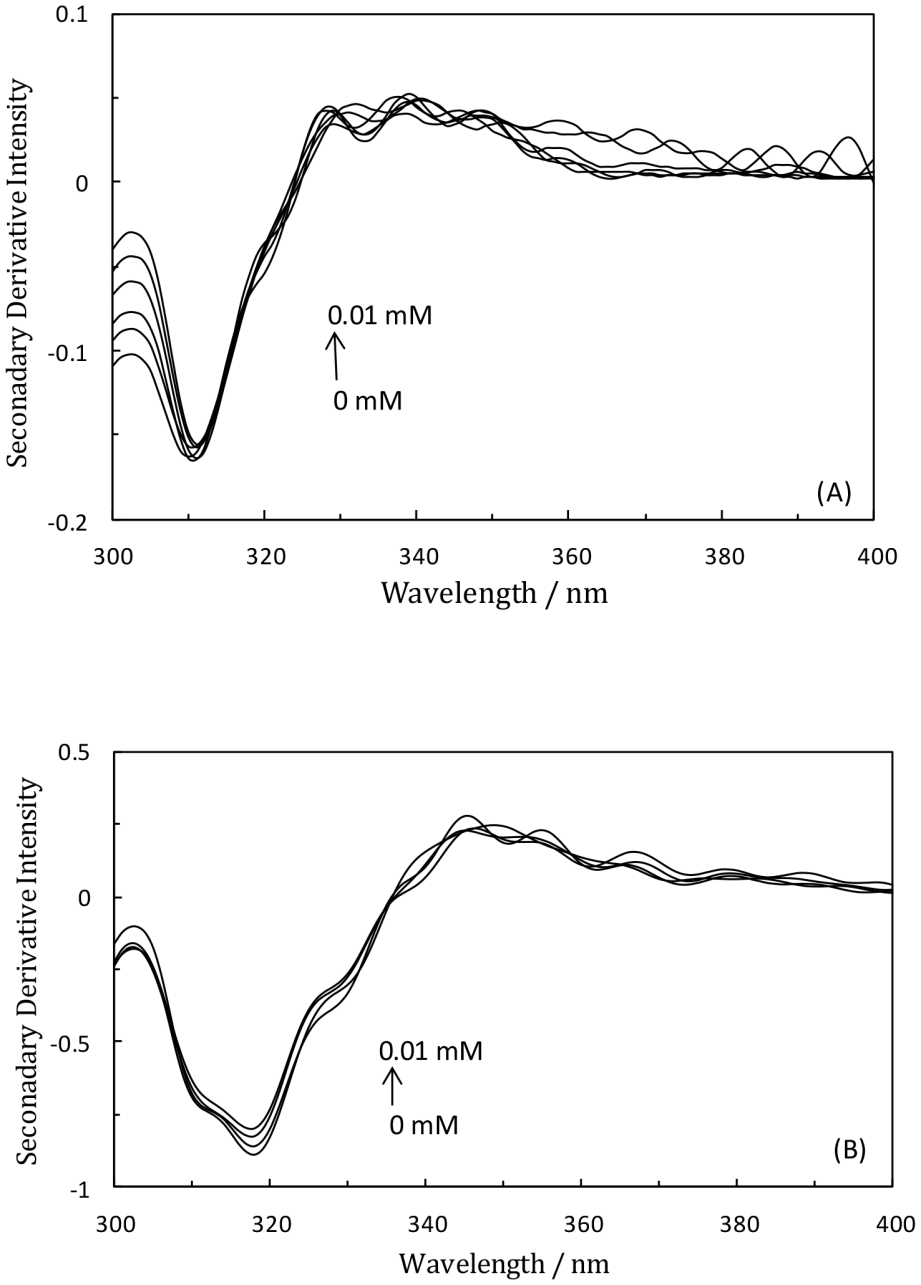
Second derivative of the Trp emission spectra of (HSA-PPIX)-LMF (A) and [(HSA-PPIX)-TF]-LMF (B). [LMF] = 0.05 mM, [(HSA-PPIX)-TF] = [HSA-PPIX] = 4.5×10^−3^ mM.

We analyzed the second derivative fluorescence of titration of (HSA-PPIX)-LMF and [(HSA-PPIX)-TF]-LMF to better understand the effect of TF on the binding of LMF to the (HSA-PPIX)-TF complex. The sensitivity of the fluorescence technique was increased by second derivative fluorescence spectroscopic which made it possible to monitor small changes taking place in the environments of aromatic amino acids in proteins [31], [32]. Fig. 3 shows second-derivative fluorescence spectra of the (HSA-PPIX)-LMF and [(HSA-PPIX)-TF]-LMF complexes (inset). As can be seen, a negative band was observed at 310 nm and 316 nm in the (HSA-PPIX)-LMF and [(HSA-PPIX)-TF]-LMF spectra, respectively. These changes in the protein spectra should be assigned to the combination of environments of the aromatic residues in the protein and indicate that the Trp residue was in a relatively hydrophobic microenvironment.

### Phase diagram method

**Figure 4:**
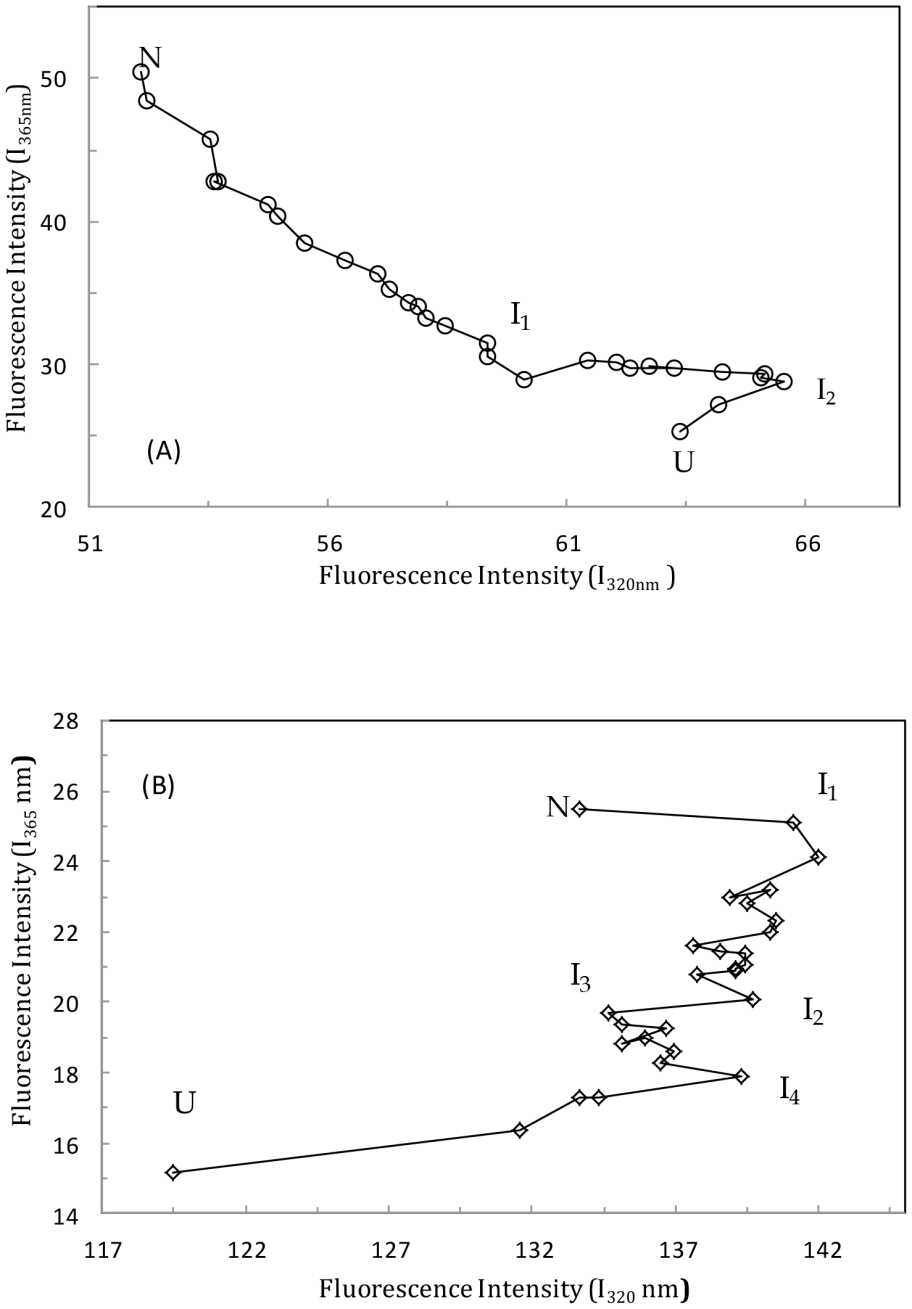
Phase diagram for the (HSA–PPIX)-LMF system (A) and the [(HSA-PPIX)-TF]-LMF system (B). [LMF] = 0.05 mM, [(HSA-PPIX)-TF] = [HSA-PPIX] = [TF] = 4.5×10^−3^ mM. λ_ex_ = 295 nm; pH 7.4, T = 298K.

Giving additional support to the idea that TF-induced unfolding of HSA-PPIX is an exceptionally complex process, Fig. 4 shows the results of the phase diagram method (see Section 2). It should be noted that applying this method to cases of protein unfolding gives the prediction that the dependence of I(V_1_)  = f(I(V_2_)) is linear if changes in the protein environment lead to an all-or-none transition between two conformations. On the other hand, non-linearity of this function reflects the sequential character of structural transformations. Moreover, each linear portion of the I (V1) = f (I(V2)) dependence describes individual all-or-none transitions. The phase diagram for (HSA-PPIX)-LMF is shown in Fig. 4A and displays three linear parts reflecting the existence of at least three independent transitions: N ↔ I_1_, I_1_ ↔ I_2_, and I_2_ ↔ U [25]. Fig. 4B shows five linear parts of the phase diagram of [(HSA-PPIX)-TF] LMF, which signifies that there were at least five independent transition states during the unfolding process and suggests the existence of intermediate structures.

#### Binding parameters

For the static quenching procedure, the binding constant, K, and the number of binding sites, n, can be obtained from the following equation [33], [34]. 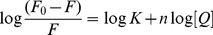
(5)


Here, [Q] is the total concentration of quencher, n can be deduced from the slope of linear regressions of the curve log (F_0_−F)/F versus log [Q], and K is determined from the intercept of the linear regression. The values of K and n are presented in Table 1. It can be seen that in the [(HSA-PPIX)-TF]-LMF complex, the n value was less than the algebraic average of n for TF-LMF and (HSA-PPIX)-LMF, separately. This difference suggests that the accessibility of the drugs to these binding sites may be limited and overlap in the presence of TF through the formation of a protein-protein complex.

The value of K illustrated that the binding affinity of LMF to (HSA-PPIX) was high. On the other hand, it was found that the binding constant of LMF to HSA-PPIX in the presence of TF decreased, resulting in a reduction of the stability and indicating that the binding interaction between LMF and the (HSA-PPIX)-TF complex was weak. Nevertheless, at high concentrations of LMF, the K value increased.

HSA is known to have three structurally homologous domains, i.e., I, II, III, each one composed of two smaller sub-domains, i.e., A and B. In our previous work [18], we showed that PPIX occupied site I in the HSA and LMF bound to site IIA. Moreover, K_sv_ for this interaction was 9.65×10^3^ M^−1^. In this study, on the other hand, in the presence of TF, the value decreased to 2.36×10^3^ M^−1^ wherefore we believe that the lower-affinity site of LMF, corresponding to the binding site, was switched in the presence of TF, causing LMF to bind to site III in HSA. It is suggested that the second binding site in the (HSA-PPIX)-TF complex was situated in TF, since the binding affinity of LMF to this site was the same as the affinity of LMF to the binding site in TF [19]. (The first and second binding sites for LMF in the (HSA-PPIX)-TF complex was determined by molecular modeling and site probe studies.).

### Site probe studies

Sudlow et al. [35] have suggested two main distinct binding sites (sites I and II) in HSA. Site I of HSA has affinity for warfarin, phenylbutazone, etc., and site II for ibuprofen, flufenamic acid, etc. It has been reported that digitoxin binding is independent of sites I and II [36]. To determine the binding site in (HSA-PPIX)-TF for LMF, site probes such as warfarin, ibuprofen and digitoxin were used to perform competitive binding studies. To do so, emission spectra of mixtures of LMF, (HSA-PPIX) and site probes were recorded separately.

The corresponding binding constants were evaluated and are listed in Table 3. The binding constant of LMF with (HSA –PPIX)-TF decreased remarkably in the presence of digitoxin, whereas it remained almost constant in the presence of warfarin. Based on these results, it was concluded that ibuprofen had little effect on the binding of LMF to (HSA-PPIX)-TF while digitoxin removed LMF from the bindings site. Moreover, the LMF binding was determined to be independent of sites I and II of HSA. In our previous work [19], it was found that LMF bound to site IIA of HSA in the presence of PPIX. Site marker experiments showed that in the presence of TF, the binding site of LMF on HSA-PPIX changed from Sudlow's site II to Sudlow's site III.

**Table 3:** Comparison of the binding constants of the (HSA-PPIX) LMF and [(HSA-PPIX)-TF] LMF systems before and after the addition of the site probe.

System	K_SV_, without the site probe (M^−1^)	K_SV_, with warfarin (M^−1^)	K_SV_, with ibuprofen (M^−1^)	K_SV_, with digitoxin (M^−1^)
(HSA-PPIX) LMF	9.65×10^3^	8.33×10^2^	9.73×10^3^	9.59×10^3^
	2.29×10^4^	2.03×10^3^	2.17×10^4^	1.43×10^4^
[(HSA-PPIX) LMF] TF	2.36×10^3^	2.25×10^3^	1.71×10^2^	2.05×10^2^
	1.08×10^4^	1.22×10^4^	8.13×10^2^	7.09×10^2^

### Three-dimensional fluorescence spectroscopy

**Figure 5:**
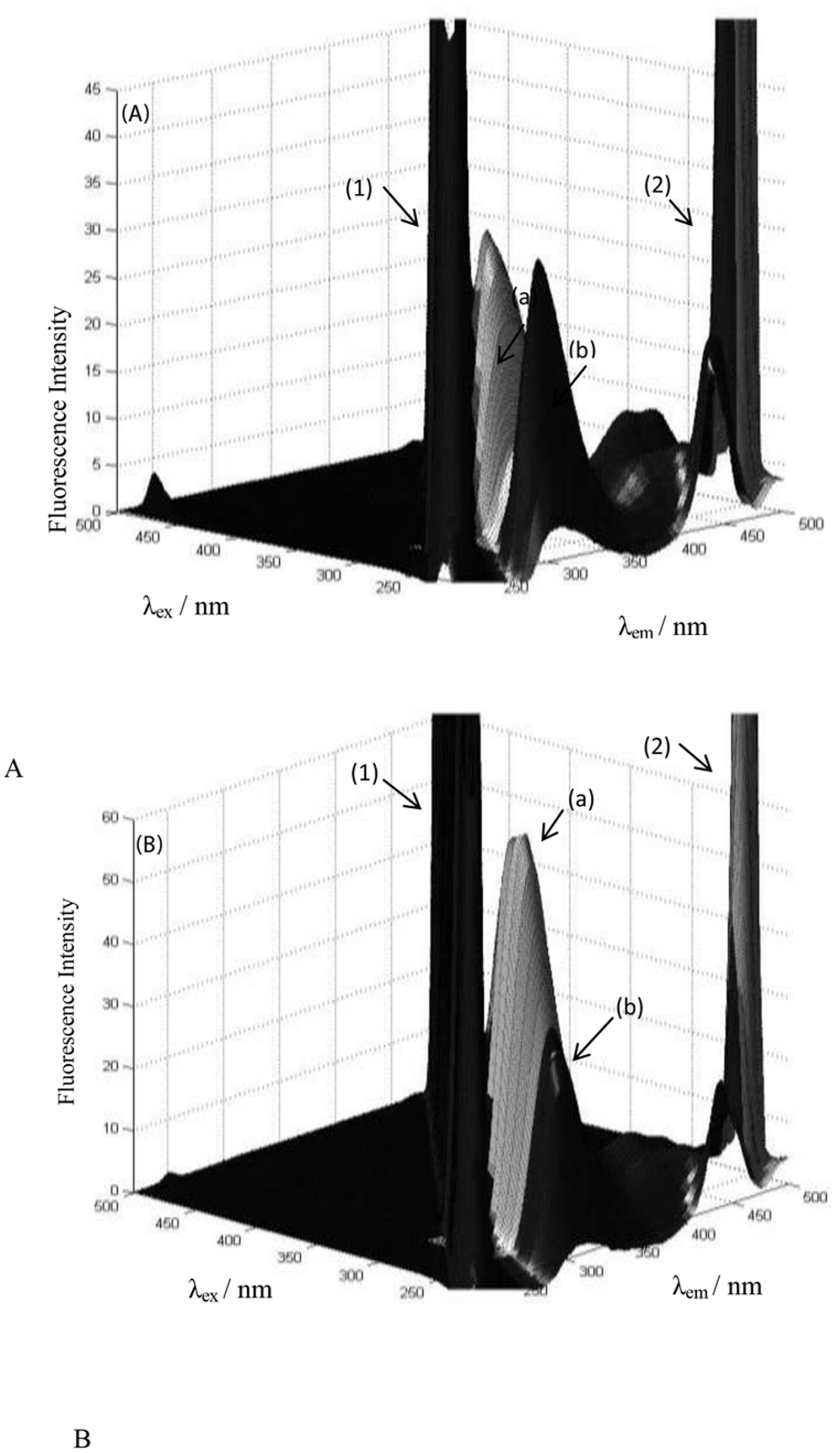
Three-dimensional fluorescence spectra of [(HSA-PPIX)-LMF], (A) and [(HSA-PPIX)-TF]-LMF, (B). [LMF] = 0.05 mM, [(HSA-PPIX)-TF] = [HSA-PPIX] = 4.5×10^−3^ mM.

Three-dimensional fluorescence spectroscopy is a new analytical technique that has been used in recent years to study the conformational changes of proteins [37], [38]. The excitation wavelength, the emission wavelength and the fluorescence intensity can be employed to investigate the characteristic conformational changes of proteins in a more scientific and credible manner [39], [40]. The three-dimensional fluorescence spectra of the HSA-PPIX and (HSA-PPIX)-TF complexes in the presence LMF are presented in Fig. 5A and B, respectively and the corresponding characteristic parameters are listed in Table 4. As shown in Fig. 5, peak 1 (λ_ex_ = λ_em_) is the Rayleigh scattering peak, and peak 2 (λ_ex_ = 2λ_em_) is the second-order scattering peak. Peak a (λ_ex_ = 280 nm, λ_em_ = 320 nm) represents the fluorescence peak and mainly enunciates the spectral behavior of the Tyr and Trp residues. In comparison with peak a, another fluorescence peak b (λ_ex_ = 235 nm λ_em_ = 340 nm) corresponds to the second-order fluorescence peak, which is mainly caused by the transition of π → π* of characteristic polypeptide backbone structure C = O of TF and HSA-PPIX [41].

**Table 4:** Three-dimensional fluorescence spectra characteristic of the interaction of (HSA-PPIX)-TF in the absence and presence of LMF.

System	Peak a			Peak b		
	λ_ex_/λ_em_	Intensity	Δλ(nm)	λ_ex_/λ_em_	Intensity	Δλ(nm)
(HSA-PPIX)-LMF	280/307	33.5	27	235/308	31.5	71
[(HSA-PPIX)-TF]-LMF	275/320	64.5	45	240/316	25.02	71

It is obvious from the figure that both fluorescence peaks a and b of these proteins have been quenched by LMF, but to different extents, as shown in Table 4. This implies that the peptide chain structure was changed and that a complex was possibly formed between LMF and the proteins thus altering the conformation. The decrease in intensity of peaks a and b suggests that the binding of LMF to HSA-PPIX induced a mild denaturation of the polypeptide chain, which led to conformational changes and revealed certain hydrophobic regions that had been buried in the [(HSA-PPIX)-TF]-LMF complex. An increase of the intensity of emission peak a signified that there occurred protein-protein interactions. Peak b is related to the backbone of the protein and decreased in the presence of LMF thus pointing at conformational changes in the protein, which supported the results of the fluorescence data.

#### Zeta potential

**Figure 6:**
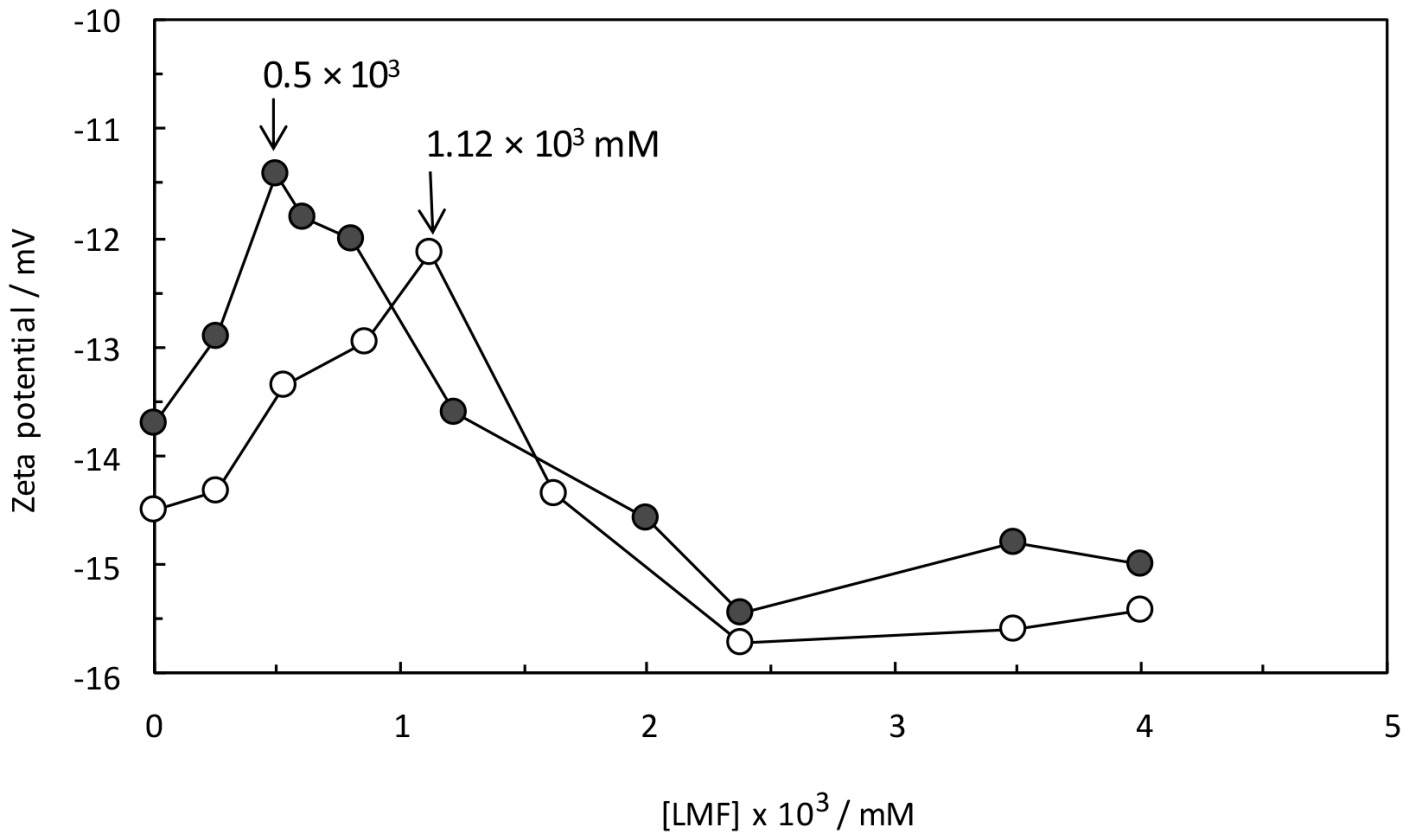
Effect of LMF on the zeta potential of (HSA-PPIX)-LMF (open circle), and [(HSA-PPIX)-TF]-LMF (filled circle) in pH 7.4.

Variation in nano-particle surface charges can control their electrostatic interaction and can be explored by measuring the zeta potential of these particles [42]–[44]. In the presence of LMF, according to Fig. 6, a comparison between HSA-PPIX and (HSA-PPIX)-TF indicated that TF caused an increase in positive charges of the HSA-PPIX complex. The surface charge of proteins arose primarily from ionization of the surface groups. The change in zeta-potential zone started close to the critical induced aggregation concentration (C_CIAC_) of the HSA-PPIX and (HSA-PPIX)-TF complex, suggesting that the protein surface was saturated. We thus hypothesized that the TF complex was involved in a certain neutralization of the net protein charge and that a conformational change of the protein allowed the C_CIAC_ point to be achieved at a lower concentration of LMF. The change in zeta potential was associated with chain entanglements, and therefore, the system became incipiently instable. This hypothesis was in agreement with important losses of tertiary structure, which led to conformational changes as observed in the results of the fluorescence measurements.

The zeta potential method can also be used to determine the polydispersity index (PDI), which is the mass average degree of the molecular weight to the number average degree of the molecular weight. The size distribution was given by the PDI (a value between 0 and 1) [45], [46]. This finding shows that the interaction of LMF with the HSA-PPIX and (HSA-PPIX)-TF complexes did not denature the proteins and a high PDI (0.5) in the finished product was ensured.

#### Molecular modeling

Molecular modeling was carried out to complement the experimental data. The literature has numerous reports of LMF being located within an α-helix and a β-sheet in the N-lobe of transferrin [46]. Analyzing the docking results for HSA-PPIX suggested that domain IIA was the most plausible site of interaction [19]. It has been reported that, in a complex of (HSA-PPIX)-TF, the LMF binding is independent of sites I and II. Rather, LMF binds to site IIIB.

**Figure 7:**
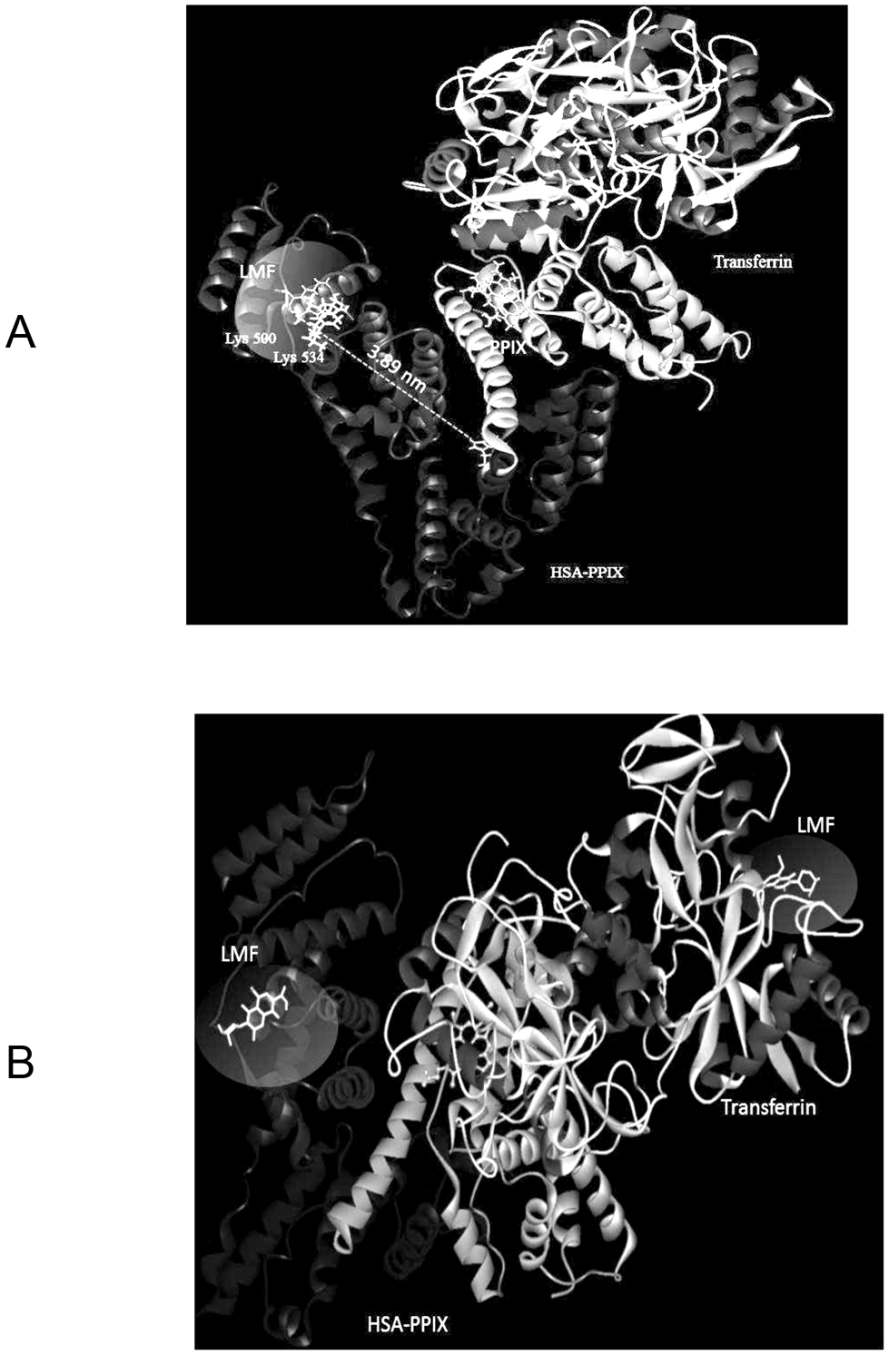
Molecular modeling of the interaction of LMF with the [(HSA-PPIX)-TF] complex, (A) and the second site of the interaction of LMF with [(HSA-PPIX)-TF] (B), represented as a solid ribbon, colored by secondary structure, LMF represented as sticks. The docking position of LMF to the protein is highlighted. LMF was docked in sub-domain IIIB of (HSA-PPIX). The distance between the binding site candidates of LMF to Trp is also illustrated. The hydrogen bonds between LMF and (HSA-PPIX) are represented as green dashed lines.

The best docking energy result for the complex of (HSA-PPIX)-TF is exhibited in Fig. 7A. LMF was located within subdomain IIIB in HSA, approximately 3.58 nm from the fluorescence of Trp 214. This LMF binding site is included in a hydrophobic cleft walled by the following amino acids: Lys, Val, Pro, Tyr, Ser, Thr, Asp, suggesting the existence of a hydrophobic interaction between them. Furthermore, there occurred hydrogen bonding between the atoms O_23_ and O_22_ of LMF with the residues Lys500 and Lys534 of HSA-PPIX. The results of the molecular modeling show that hydrophobic forces and hydrogen bonds were responsible for the interaction between LMF and the (HSA-PPIX)-TF complex. In order to investigate the second binding site for LMF in the (HSA-PPIX)-TF complex, [(HSA-PPIX)-TF]-LMF was subjected to energy minimization with an MM^+^ force field after which the best docking results were used for further analysis. As depicted in Fig. 7B, LMF bound to the N-lobe of TF. These results agree fairly well with those of the spectroscopic and zeta potential studies.

## Conclusion

To study the effect of TF on the binding of LMF to a (HSA-PPIX) complex, fluorescence spectroscopic, zeta potential and molecular modeling techniques were used. Quantitative analysis of the protein structure by fluorescence spectroscopy showed that conformational changes in HSA-PPIX were induced by TF, and that they could alter the affinity of this complex to other ligands. It was concluded that certain binding sites overlapped with each other in the (HSA-PPIX)-TF complex, suggesting that the accessibility of drugs to these binding sites may be limited in the presence of TF. It is therefore important, especially in the case of cancer, to increase the amount of PPIX in the blood, and follow this we have amount of HSA-PPIX and also (HSA-PPIX)-TF complex.

For the (HSA-PPIX)-TF complex in solution, the binding site of LMF to HSA-PPIX changed from Sudlow's site IIA to Sudlow's site IIIB with low affinity. For a second binding site at high concentrations of LMF, the binding site with the highest affinity was located in TF. Changes in binding site of proteins play a major role for drug delivery capability. The presence of TF gives rise to an increase of the positive charges of the HSA-PPIX complex, as demonstrated by zeta potential measurements, causing protein conformational changes. This phenomenon can be explained by the partial neutralization of HSA-PPIX ions by the positive charge of TF. The PDI results obtained showed that a complex of LMF with these proteins did not denature the proteins. Molecular modeling of the complex (HSA-PPIX)-TF pointed at LMF being located within sub-domain IIIB in HSA and the N-lobe of TF.

In summary, this study provides useful information for the pharmacological and pharmacodynamic behavior of LMF and should be taken into account when calculating its dosage.
